# Human bite injuries in the oro-facial region at the Muhimbili National Hospital, Tanzania

**DOI:** 10.1186/1472-6831-8-12

**Published:** 2008-04-30

**Authors:** Farrid M Shubi, Omar JM Hamza, Boniphace M Kalyanyama, Elison NM Simon

**Affiliations:** 1 Department of Oral Surgery and Oral Pathology, School of Dentistry, Muhimbili University of Health and Allied Sciences (MUHAS) Dar es Salaam, Tanzania

## Abstract

**Background:**

Human bites in the maxillofacial region compromise function and aesthetics, resulting in social and psychological effects. There is paucity of information regarding human bite injuries in Tanzania. The aim of the study was to assess the occurrence, treatment modalities and prognosis of human bite injuries in the oro-facial region at the Muhimbili National Hospital Dar es Salaam, Tanzania.

**Methods:**

In a prospective study the details of patients with human bite injuries in the oro-facial region who attended at the Department of Oral and Maxillofacial Surgery of the Muhimbili National Hospital between January 2001 and December 2005 were recorded. Data included information on age, sex, site, duration of the injury at the time of reporting to hospital, reasons, details of treatment offered and outcome after treatment.

**Results:**

A total of 33 patients, 13 males and 20 females aged between 12 and 49 years with human bite injuries in the oro-facial region were treated. Thirty patients presented with clean uninfected wounds while 3 had infected wounds. The most (45.5%) frequently affected site was the lower lip. Treatment offered included thorough surgical cleansing with adequate surgical debridement and primary suturing. Tetanus prophylaxis and a course of broad-spectrum antibiotics were given to all the patients. In 90% of the 30 patients who were treated by suturing, the healing was uneventful with only 10% experiencing wound infection or necrosis. Three patients who presented with wounds that had signs of infection were treated by surgical cleansing with debridement, antibiotics and daily dressing followed by delayed primary suturing.

**Conclusion:**

Most of the human bite injuries in the oro-facial region were due to social conflicts. Although generally considered to be dirty or contaminated they could be successfully treated by surgical cleansing and primary suture with a favourable outcome. Management of such injuries often need multidisciplinary approach.

## Background

Human bite injuries are usually considered as those injuries that occur because of a person being bitten by another person [1]. Commonly, human bites by an assailant occur extra-orally but in few occasions they also occur intra-orally. The size and severity of the injuries vary, ranging from small lacerations, punctures or cuts to total avulsion and loss of relatively big chunks of tissue [2-4]. Because of their location, human bites in the maxillofacial region compromise function and aesthetics, as a consequence social and psychological effects are most likely.

In the USA, one in every two persons will be bitten in his/her lifetime by either an animal or another human being [5]; the human bites being the third most common following those of dogs and cats. In Papua New Guinea the commonest cause of bite injury was human [6]. Studies conducted in Ghana, Zimbabwe and Nigeria have shown that in most incidences the assailant is a person known to the victim [2,7-9]. However, in these studies there existed differing findings regarding demographic data of the victims and the basic reasons behind the attacks.

Human bite injuries are generally considered to be dirty or contaminated [1]. These wounds have been exposed to the attacker's oral microflora and, for intra oral wounds, to both the victim's and the attacker's oral microflora. Surgically they pose a challenge, because of the tenet that human bite wounds should always be handled as contaminated wounds since the risk of infection is high, especially in situations where there is delay in reporting for medical care. Nevertheless, due to local circumstances, and following findings and suggestions from different authors [2,8,10], it is a common practice in the Oral Surgery Department at the Muhimbili National Hospital (MNH), to primarily suture the wounds after thorough surgical cleansing and debridement regardless of time elapsed after injury, so long as clinically there is no evidence of infection. However, in Tanzania there has never been any analysis to determine the magnitude of the problem of human bite injuries in the facial region and prognosis of patients treated as such.

Therefore, it was the aim of this study to determine the occurrence, treatment modalities and prognosis of human bite injuries in the oro-facial region, at the Oral Surgery Department of the Muhimbili National Hospital (MNH).

## Methods

### Participants and setting

This study was conducted at the MNH during a period of five years (January 2001 – December 2005). All patients who suffered human bite injuries in the facial region were included in the study.

### Study design and methodology

Patients' demographics (age, sex, occupation, marital status), interval from injury to reporting to hospital, site, condition of the wound and treatment were recorded in a special form. After a thorough clinical examination the injuries were classified into superficial or deep, and infected or non-infected. A wound was considered to be superficial if it did not dehisce and infected if it showed signs of presence of pus.

### Treatment

**Figure 1:**
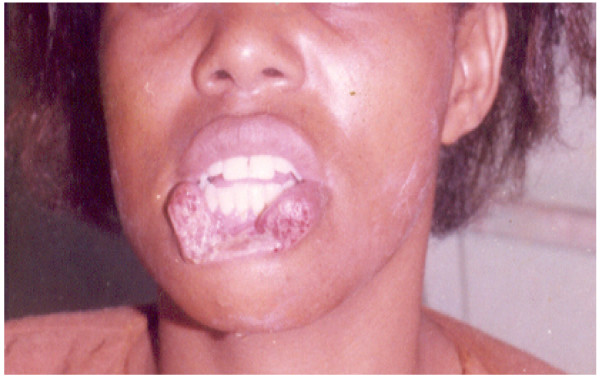
Human bite injury of the lower lip with avulsion of tissue.

**Figure 2:**
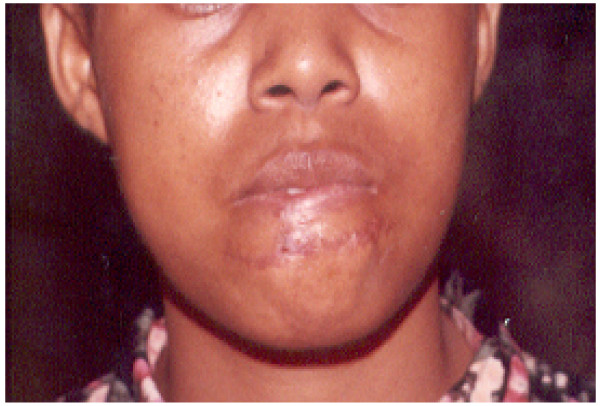
The patient four weeks after treatment by primary suture.

**Figure 3:**
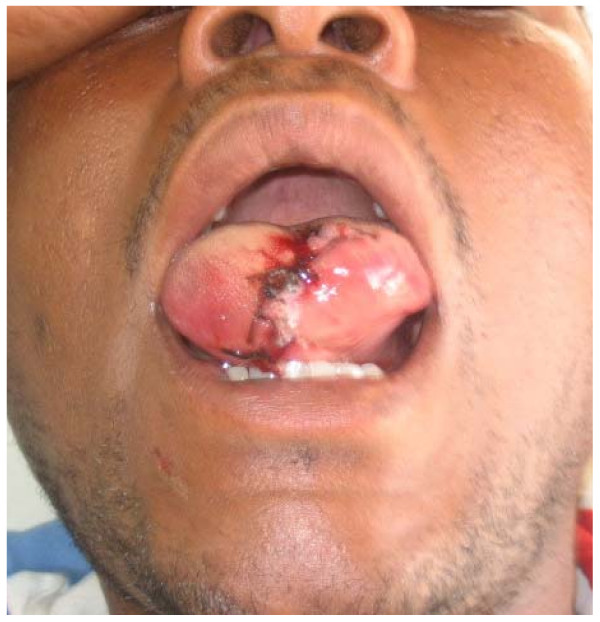
Patient with human bite injury of the tongue immediately after primary suture.

For wounds that were not infected, treatment included thorough surgical toilet, suturing, antitetanus and broad spectrum antibiotics. Ampicillin/amoxicillin were used in combination with metronidazole and in some cases either ciproflaxin or augmentin were used. Surgical cleansing involved debridement to remove all debris and non-vital tissue and thorough irrigation with 3% hydrogen peroxide followed by sterile normal saline. Edges were approximated and using vicryl and silk or proline suture materials wounds were sutured in layers to abolish dead space. Smaller suture materials (silk 4.0) were used for suturing the vermilion border of the lip. In two cases where big portions of the lower lip were avulsed local advancement flaps were raised bilaterally to reconstruct the lip to achieve desirable functions and aesthetics (Fig. 1 and Fig. 2). In two cases where the anterior one third of the tongue was completely severed (Fig. 3), undermining of the tissues was done and wounds sutured in layers. The edges of the dorsum and ventral surfaces were then approximated and sutured. Three cases that presented with signs of infection were not sutured primarily but rather thorough surgical cleansing and debridement were performed, followed by dressing with antiseptics and administration of systemic broad-spectrum antibiotics. Sutures were normally removed after seven days. Delayed primary suturing was done only after the wounds appeared clean without suppuration or discharge.

### Ethical issues

Ethical clearance was granted by the Muhimbili University of Health and Allied Sciences (MUHAS) research and publications committee and permission to conduct the study was given by the Muhimbili National Hospital.

Informed verbal consent was obtained from all the patients. The aims and benefits of the study were elaborated to the participants. They were also informed that acceptance or rejection to participate would not affect the quality of care they would receive. All information provided by the patients was kept under strict confidentiality.

### Statistics

Data were fed in computer and frequencies calculated for comparisons.

## Results

A total of 33 patients aged between 12 and 49 years presented with human bite injuries in the facial region (Table 1). There were 13 males and 20 females with a male to female ratio of 0.6:1.

**Table 1:** Human bite injuries by sex and age

Age	Sex				Total	
			
	Male		Female			
	n	(%)	n	(%)	n	(%)
12 – 19	1	(3)	3	(9.1)	4	(12)
20 – 29	5	(15.2)	10	(30.3)	15	(45.5)
30 – 39	4	(12)	5	(15.2)	9	(27.3)
40 – 49	3	(9)	2	(6)	5	(15.2)

Total	13	(39.4)	20	(60.6)	33	(100)

Majority, 24 (72.7%) patients reported to hospital during the first 24 hours. The most commonly involved site was the lower lip 15 (45.5%), followed by the ear 4 (12.1%) (Table 2). Thirty patients presented with wounds that were clinically free of infection while three had wounds that showed signs of infection. The patients with infected wounds presented 36 hours or more after injury. Over 70% of the injuries occurred after dusk. Except for 8 (24.2%) cases, all the other 25 (75.8%) victims knew the assailants (Table 3). None of the patients presented avulsed parts to hospital.

**Table 2:** Distribution of human bite injuries by site and sex

Site	Sex				Total	
			
	Male		Female			
	n	(%)	n	(%)	n	(%)
Lower lip	4	(12.1)	11	(33.3)	15	(45.5)
Upper lip	1	(3)	2	(6.1)	3	(9.1)
Nose	2	(6.1)	1	(3)	3	(9.1)
Tongue	3	(9.1)	-		3	(9.1)
Ear	2	(6.1)	2	(6.1)	4	(12.1)
Tongue + lower lip	-		1	(3)	1	(3)
Upper lip + Nose	1	(3)	-		1	(3)
Cheek	-		3	(9.1)	3	(9.1)

Total	13	(39.4)	20	(60.6)	33	(100)

**Table 3:** Distribution of the patients according to the kinds of people who attacked them.

Attacker	Male		Female		Total	
	n	(%)	n	(%)	n	(%)
	
1. Wife	2	(6)	-	-	2	(6)
2. Co-wife (jealousy)	-	-	3	(9)	3	(9)
3. Closely related (work, social disagreements....)	4	(12)	10	(30)	14	(42.4)
4. Known to victim but not related	1	(3)	5	(15)	6	(18)
5. Unknown to victim (robberies/attempted robberies)	6	(18)	2	(6)	8	(24)

Total	13	(39.4)	20	(60.6)	33	(100)

### Treatment outcome

Out of thirty cases that were treated primarily on presentation, twenty-seven (90%) healed uneventfully with desirable aesthetic effects while three (10%) patients had infections of the wounds with some necrosis of the involved tissues. All of the three patients who were treated by delayed suturing after clearing the infection ultimately had good healing.

## Discussion

MNH is the largest centre where all cases that need specialized treatment from all dispensaries, health centres and municipal hospitals in the city of Dar es Salaam are referred to. The present study exhibits an interesting sexual bias, showing a higher frequency in females compared to males. It is in agreement with the studies from Zimbabwe and Nigeria, which showed that most attackers and victims were females but is in disagreement with results from Ghana where more males than females were involved [1,8,9,11]. In time of emotion and anger the teeth might have appeared to be the most readily available weapon to be used during attack or defence. Also, females might have preferred to use teeth during fights as quite often the fights were related to jealousy therefore might have wished to effect maximum harm to their "enemies" [4].

Majority of the victims were aged between twenty and thirty nine years with the peak incidence in the 20 to 29 years age group. This is most possibly so because the social activities of the young section of the population often lead to conflicts that might end up to be settled through physical means. Most of the cases presented to hospital after dusk which might be explained by the fact that it is usually at the end of the day that people indulge in activities like alcohol drinking, music and dancing or unlawful acts like robberies that might have eventually led to fights (attacks or self defence) (Table 3). The reasons behind the occurrence of human bite injuries as cited in different studies are complex [7,9,10,12]. Eardly et al. (2006) showed a clear link between weekend drinking and human bite injuries in North East England [12]. Although the present study is based on a different cohort, the reasons mentioned by the patients we saw are remarkably similar to those reported from Nigeria, Zimbabwe and Ghana [7,9-11]. Matrimonial conflicts in mono or polygamous marriages featured prominently among the reasons for fights between man and wife or wife and co-wife. This reflects the life-style of people in Tanzania, a multicultural and secular country with different cultures and religions, some of which support polygamous marriages. Other reasons that were cited included jealousy, unfaithfulness in marriage and disagreements in family matters or at work places (Table 3). Robberies or attempted robberies of personal possessions, which featured in this study, hardly appeared in any of previous African studies. Such acts were most possibly precipitated by an increase in unemployment in the city due to a rural to urban migration by youth seeking a better life and redundancies that followed collapse of many government parastatals and/or privatisation of the economy.

The lower lip was the site that was most (45.5%) commonly involved (Fig. 1a), followed by the ear (12.1%). This could be explained by the position of the lower lip in the face, which makes it among the most prominent parts in the face and therefore easy to be grabbed by the attacker's teeth. These findings are in agreement with those of Obukwe (2002) in Nigeria who also found the lower lip to be the most commonly involved site [8]. For the two cases where considerably big portions of the tongue were severed (Figure 2), it is intriguing to imagine how the attacker's teeth got access so deep in the mouth to reach the tongue, much so because both victims were fully dentate. In situations like these, where the tongue is amputated, it poses great surgical challenges considering the specialized functions of the tongue. A systematic search in the available literature could not assist because hardly any cases of severed tongue by human teeth have been reported.

Some patients who had wounds that involved the nose were treated in cooperation with the otorrhinolaryngologist. Such multidisciplinary approach offered the patients an added advantage of achieving maximum aesthetics and functional effects. Among factors that must be considered in deciding treatment include cosmetic significance, local blood supply and other general host factors.

Empirically infection is expected in animal bite cases including human bites. The fact that most (90%) of the patients had good healing of the wounds after primary suture partly was because of the good blood supply in the facial region which increases the host resistance in the local area and the choice of antibiotics. The infection rate as seen in our study is nearly similar to the 8.3% reported by Donkor and Bankas (1997) in Ghanaian patients [9]. Taking into consideration the nature and multitude of the resident microflora in the oral cavity, the antibiotics chosen must always be those with broad-spectrum activity.

Despite the diversity of opinion regarding the approach to management of human bite injuries in the facial region as expressed by others [1,10-13], it is our conviction that the findings of the present study lends strong support to primary closure for all human bite injuries that present without signs of infection regardless of the time lapsed since injury. Baurmash and Moto (2005) attempted with success primary closure in cases that had obvious signs of infection [14]. However, in their study they applied hyaluronidase, which is believed to take care of the oedema that is empirical in the immediate pre and post surgical period. Similar to some of the cases that were attended to in the present study which were quite extensive with losses of tissue, Agrawal et al. (1992) advocates primary closure in all human bite wounds regardless of the size [4]. In their study the management of majority of their cases by primary closure proved highly successful.

This deviation from the norms of general surgery, which has shown good results, offered the following advantages:

The patients who were residing far away from the hospital did not have the trouble of visiting the hospital daily for dressing.

The anxiety of the possibility of a second operation for delayed primary suture or secondary suture was minimized.

Furthermore, this resulted in a significant cost reduction to both the patient and the institution, which are important considerations in the generally poor Tanzanian population.

## Conclusion

Most of the human bite injuries in the oro-facial region were due to social conflicts in the community. Although generally considered to be dirty or contaminated, human bite injuries could be successfully treated by thorough surgical cleansing and primary suture with a favourable outcome. Human bite injuries in the oro-facial region often need multidisciplinary approach in their management. We recommend that all human bites without obvious signs of infection should be closed primarily. Public education on complications that might arise from human bite injuries would minimize their incidence and encourage the victims to report early for treatment.

Boosting security in the urban centres by installation of streetlights and increasing police patrols and reduction of unemployment and curtailing the rural to urban youth migration are other measure that could reduce the roblem.

## Competing interests

The authors declare that they have no competing interests.

## Authors' contributions

All authors actively participated in designing the study. FMS, BMK and ENMS treated the patients. OJMH collected data and performed the data analysis. All authors took part in preparations of the manuscript. They all read and approved the manuscript for processing for submission for publication.

## Pre-publication history

The pre-publication history for this paper can be accessed here:


